# Sodium Ferulate Inhibits Neointimal Hyperplasia in Rat Balloon Injury Model

**DOI:** 10.1371/journal.pone.0087561

**Published:** 2014-01-29

**Authors:** Jing Zhang, Jing Chen, Jian Yang, Changwu Xu, Jiawang Ding, Jun Yang, Qing Guo, Qi Hu, Hong Jiang

**Affiliations:** 1 Department of Cardiology, Renmin Hospital of Wuhan University, Wuhan, Hubei, PR China; 2 Department of Cardiology, The First College of Clinical Medical Sciences, China Three Gorges University, Yichang, Hubei, PR China; 3 Department of Ophthalmology, The First College of Clinical Medical Sciences, China Three Gorges University, Yichang, Hubei, China; The University of Tennessee Health Science Center, United States of America

## Abstract

**Background/Aim:**

Neointimal formation after vessel injury is a complex process involving multiple cellular and molecular processes. Inhibition of intimal hyperplasia plays an important role in preventing proliferative vascular diseases, such as restenosis. In this study, we intended to identify whether sodium ferulate could inhibit neointimal formation and further explore potential mechanisms involved.

**Methods:**

Cultured vascular smooth muscle cells (VSMCs) isolated from rat thoracic aorta were pre-treated with 200 µmol/L sodium ferulate for 1 hour and then stimulated with 1 µmol/L angiotensin II (Ang II) for 1 hour or 10% serum for 48 hours. Male Sprague-Dawley rats subjected to balloon catheter insertion were administrated with 200 mg/kg sodium ferulate (or saline) for 7 days before sacrificed.

**Results:**

In presence of sodium ferulate, VSMCs exhibited decreased proliferation and migration, suppressed intracellular reactive oxidative species production and NADPH oxidase activity, increased SOD activation and down-regulated p38 phosphorylation compared to Ang II-stimulated alone. Meanwhile, VSMCs treated with sodium ferulate showed significantly increased protein expression of smooth muscle α-actin and smooth muscle myosin heavy chain protein. The components of Notch pathway, including nuclear Notch-1 protein, Jagged-1, Hey-1 and Hey-2 mRNA, as well as total β-catenin protein and Cyclin D1 mRNA of Wnt signaling, were all significantly decreased by sodium ferulate in cells under serum stimulation. The levels of serum 8-iso-PGF2α and arterial collagen formation in vessel wall were decreased, while the expression of contractile markers was increased in sodium ferulate treated rats. A decline of neointimal area, as well as lower ratio of intimal to medial area was observed in sodium ferulate group.

**Conclusion:**

Sodium ferulate attenuated neointimal hyperplasia through suppressing oxidative stress and phenotypic switching of VSMCs.

## Introduction

Percutaneous coronary intervention (PCI) is the main therapeutic approach to coronary heart disease. Although the obvious therapeutic effects are encouraging, the clinical complications following PCI operation cannot be ignored. Neointimal formation after vascular injury is the main cause reducing cure rate and leading to rehospitalization or revascularization [1]. With the advent of drug-eluting stent, a great progress has been made in preventing restenosis [2]. Antiproliferative drugs are coated onto the stent and released slowly, interrupting cell proliferation cycle and significantly reducing in-stent restenosis [3]. However, a series of clinical consequences, including poor re-endothelialization, delayed vessel function restoration and higher cardiac risk, demonstrate that present drug-eluting stent introduction still needs careful consideration [4], [5]. Therefore, to understand the complex mechanism involved in restenosis and seek a new optimal way to attenuate neointimal hyperplasia have important clinical significance.

Current evidence indicates intimal hyperplasia after artery injury is an intricate pathological process. It consists of thrombosis, inflammation, proliferation and extracellular matrix production [6]. Immediate endothelial denudation after angioplasty results in the exposure of circulating cells to subendothelial matrix [7]. The activated platelet, numerous cytokines and reactive oxygen species (ROS) are recruited, thus accelerating thrombosis, inflammation and oxidative stress in vessel lesions [8], [9], [10]. In response to these environmental changes induced by endothelial screen deprivation, the medial vascular smooth muscle cells (VSMCs) turned from contractile to synthetic subtype. The new dedifferentiated phenotype facilitates VSMCs migration to the intima and begins to proliferate and secrete extracellular matrix, which leads to the formation of the thick neointimal after acute phase [6]. Unlike the skeletal and cardiac muscle cells, VSMCs phenotype transition is reversible and regulatory [7]. Many studies have demonstrated that phenotype modulation from synthetic to contractile shows great suppression on neointimal hyperplasia [11], [12], [13], [14].

Sodium ferulate (3-methoxy-4-hydroxy-cinnamate sodium, SF), the salt of ferulate acid, is widely distributed in Ligusticum, Chuanxiong, Propolis and other herbs [15], [16]. As ferulate acid of active ingredient, sodium ferulate is an approved clinical drug with better solubility and lower toxicity than ferulate acid [17]. Previous studies reveal sodium ferulate or ferulate acid possesses a variety of beneficial pharmacological effects on human diseases. Due to its favorable anti-inflammatory and anti-oxidant properties, ferulate acid could attenuate ischemic injuries, atherosclerosis and diabetic related damages [18], [19], [20]. The latest research suggests ferulate acid is a novel inhibitor of presynaptic glutamate release, and sodium ferulate can protect cortical neurons against glutamate toxicity [21], [22]. In addition, ferulate acid has a great capacity of anti-platelet aggregation, anti-hypertension and anti-tumor [23]. Curcumin, a derivative of ferulate acid, is an inhibitor of rabbit VSMCs proliferation [24]. However, the effect of ferulate acid or sodium ferulate on neointimal hyperplasia after vessel injury remains unclear. The aim of this study was to identify whether sodium ferulate has inhibitory effect on neointimal formation and the underlying processes.

## Materials and Methods

### Ethics statement

All rats used in present study were purchased from Animal Center of Renmin Hospital of Wuhan University. All experimental procedures were authorized by Animal Care and Use Committee of Wuhan University, and strictly complied with guidelines for the Care and Use of Laboratory Animals by the National Institutes of Health (NIH publication No. 85–23, revised 1996).

### Primary cell culture

Primary VSMCs were isolated from thoracic aortae of male Sprague-Dawley rats weighing 180-200 g. Cells were cultured in medium containing Dulbecco’s modified Eagle’s medium (DMEM, Hyclone) (Logan, Utah, USA), 10% fetal bovine serum (FBS, Hyclone), 100U/ml penicillin and 100 µg/ml streptomycin (Hyclone). The third to fifth passage cells were used for experiments.

### Cell proliferation and migration assay

VSMCs proliferation was performed with the cell counting kit-8 (CCK-8) according to the manufacturer’s protocol (Dojindo Laboratories, Kumamoto, Japan). In brief, 8,000 cells were seeded in each well of 96-well plate. After synchronous growth in DMEM including 0.5% FBS for 24 hours, VSMCs were pre-incubated in a variety of concentrations of sodium ferulate (0, 50, 100 and 200 µmol/L) (Suzhou Changtong Chemical, Suzhou, China) for 1 hour and then stimulated with Ang II (1 µmol/L) (Peprotech, CT, USA) for another 48 hours. Following addition with CCK-8 reagent, OD values were measured at 450 nm using microplate spectrophotometer.

Transwell chamber was used for detecting VSMCs migration. 10^4^ synchronized cells were seeded into the upper chamber containing 200 µmol/L sodium ferulate in 200 µL DMEM. The lower chamber was filled with 1 µmol/L angiotensin II in 600 µL DMEM. After incubation for 8 hours, non-migrated cells were removed from filter membrane. The lower membrane was fixed in methanol and stained with 0.1% crystal violet. Cells from five random fields were calculated under microscope at a magnification of 100.

### Reactive oxygen species assay

A fluorescent probe (Beyotime, Shanghai, China) was used to detect intracellular ROS level. VSMCs were seeded onto 24-well plate at a density of 1.5×10^4^(each well). Synchronized for 24 hours, cells were incubated with 200 µmol/L sodium ferulate for 1 hour and then exposed to 1 µmol/L Ang II for another 1 hour. The fluorescent probe DCFH-DA was added at a final concentration of 10 µmol/L and incubated at 37°C for 20 minutes. Pictures were taken under fluorescence microscope and the fluorescence mean density was measured from three random fields for each well.

### NADPH oxidase and SOD activity assay

Cultured VSMCs were pre-treated with sodium ferulate and induced by Ang II as described above, and NADPH oxidase activity was detected following previous study [25]. Briefly, cells were incubated in lysis buffer on ice for 30 minutes and then centrifuged at 14,000 rpm for 5 minutes. The supernatant was added to assay buffer in presence of 100 mM lucigenin and 100 mM NADPH (Sigma, MO, USA) in a final volume of 1 mL. Sample chemiluminescence was measured immediately, and the NADPH oxidase activity was expressed as units/sec.mg protein. For SOD activity, an assay kit (Cell Biolabs, CA, USA) was used according to the manufacturer’s instructions.

### Cell phenotypic transition

Following synchronization in medium with 0.5% serum for 24 hours, VSMCs were cultured with non-serum for 72 hours. Under this starved circumstance, VSMCs started to present contractile phenotype. Then 10% serum was added with or without sodium ferulate pre-treatment to promote cell phenotype to transfer into the synthetic pattern. After incubation at 37°C for another 48 hours, the evolution process of cell phenotypic transition was completed.

### Western blot analysis

The total, cytoplasmic and nuclear proteins were isolated using protein extraction kit (Beyotime) according to the manufacturer’s instructions. After quantitation (BCA kit, Beyotime), protein was separated by electrophoresis on 10% SDS-polyacrylamide gels and transferred onto PVDF membrane. Antibodies against β-actin, β-catenin, smooth muscle myosin heavy chain protein (SM-MHC), Notch1 (Santa Cruz, CA, USA), GAPDH, histone H3, phospho-p38, phospho-ERK1/2 (Cell Signaling Technology, MA, USA) and smooth muscle α-actin (SM α-actin) (Sigma) were used to probe with the interest blots. Finally, protein expression was detected with HRP-conjugated second antibody (Santa Cruz) and ECL (Pierce, IL, USA) luminescence method.

### Quantitative real-time RT-PCR

The total RNA was extracted using the picopure RNA isolation kit (Applied Biosystems, CA, USA). The purified RNA was immediately reversely transcribed into cDNA using first-strand synthesis system (Invitrogen, CA, USA). Addition with primer sequences and Sybergreen supermixt (Bio-Rad, CA, USA) in the proper proportions, purified cDNA was get into amplification. Data were normalized to GAPDH and analyzed by 2^−ΔΔCt^ method. The primer sequences used in this study were displayed in Table 1.

**Table 1:** Primer Sequences.

	Sense	Antisense
Jagged-1	5′-CGCCCAATGCTACAATCGTG-3′	5′-GGTTGCCCTCACAGTCGTT -3′
Hey-1	5′-GCCGACGAGACCGAATCAAT-3′	5′-GGAGACCAGGCGAACACGA-3′
Hey-2	5′-TTGACAGAAGTGGCGAGGTA -3′	5′-ATGGCGTTGACTCTGATGTG-3′
Cyclin D1	5′-CTGCTGGCGAAGGTTTAGGG -3′	5′- GGAGCGGCGGCAAGAATG -3′
GAPDH	5′-GACATGCCGCCTGGAGAAAC-3′	5′-AGCCCAGGATGCCCTTTAGT-3′

### Balloon injury model in rat carotid artery

Thirty-six male Sprague-Dawley rats weighing 350–400 g were randomly divided into three groups: sham group, saline group and sodium ferulate group (n = 12 for each group). Animals were anesthetized via intraperitoneal injection with 2% sodium pentobarbital (Sigma) at a dose of 40 mg/kg. The common, internal and external carotid arteries were exposed sequentially. A balloon catheter (balloon diameter 1.25 mm, length 15 mm; Medtronic, MN, USA) was introduced into the common carotid in the case of systematic heparinization (100 U/kg, intravenous injection). The balloon was inflated and passed three times with rotation. Rats in sham group underwent the same procedure, except for artery injury. Animals in sodium ferulate group were administrated with 200 mg/kg sodium ferulate through intragastric way once a day, from the first day after surgery. Rats in saline group were taken in equal volume saline as control. All animals were fed with conventional diet until sacrificed.

### Serum 8-iso-PGF2α detection

Blood samples from external jugular vein were collected 7 days after injury, when rats were sacrificed. After centrifugation at 3000 rpm for 10 min, the upper serum were extracted and stored at –80°C. An ELISA kit (Cell Biolabs) was used to quantify serum 8-iso-PGF2α according to manufacturer’s instructions.

### Histomorphological analysis

At the 7th day, the common arteries on operated side were collected and fixed in 4% paraformaldehyde. After embedded in paraffin, arteries were cut into sections of 4 µm thickness and stained with hematoxylin and eosin. The lumen, neointimal and medial areas were measured and the ratio of intimal to medial area was also calculated by image analysis software (Image Pro Plus 6.0). Immunofluorescence assay and Masson trichrome staining were also used to evaluate vessel sections.

### Statistical analysis

All statistical analysis was performed with Statistical Product and Service Solutions 13.0 software (SPSS 13.0). Data was represented as means ± SD. Statistical analysis was performed by Student-Newman-Keuls post-test for comparison between two groups, and one-way ANOVA for multiple groups. *P*<0.05 was considered as statistically significant.

## Results

### Sodium ferulate inhibits Ang II induced VSMCs proliferation and migration

**Figure 1:**
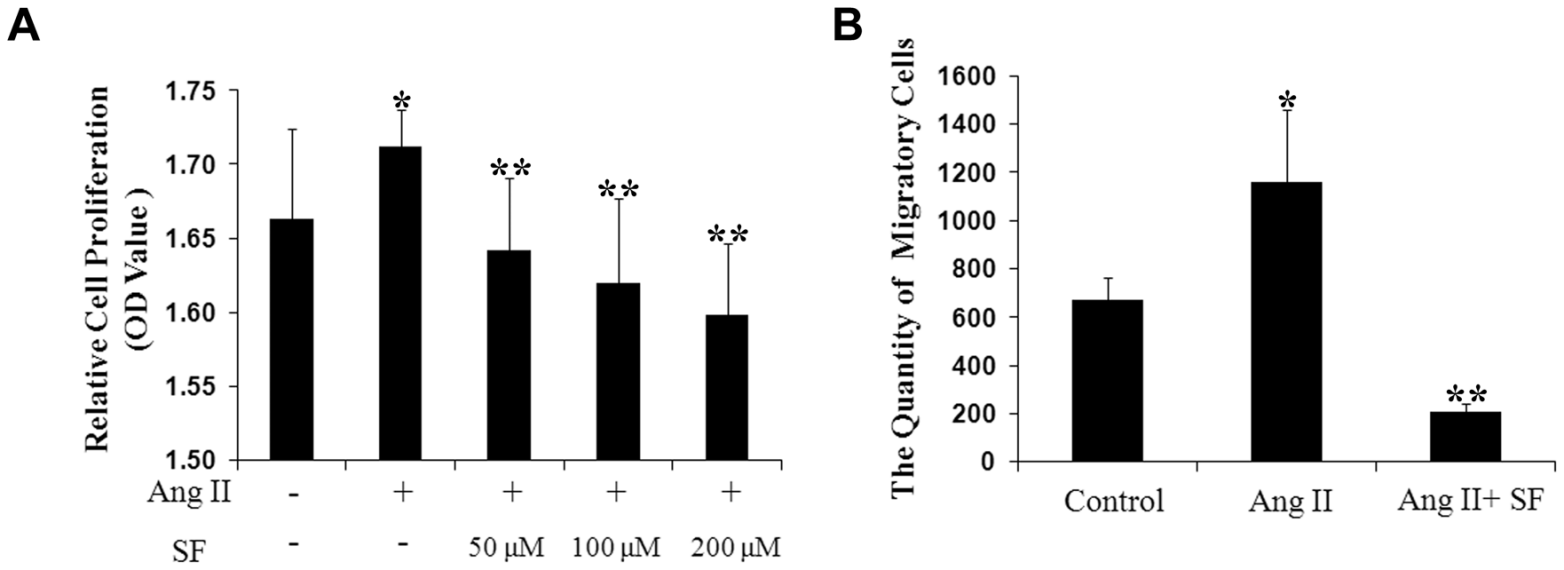
Effect of sodium ferulate on VSMCs proliferation and migration under 1 µmol/L Ang II stimulation. VSMCs were pre-incubated with sodium ferulate in a range from 50 to 200 µmol/L for 1 hour and stimulated with 1 µmol/L Ang II for the indicated time. (A) Cell proliferation was quantified by CCK-8 kit (n = 6). (B) Cell migration was performed with transwell method (n = 3). ^*^ *P*<0.05 vs. the control group, ^* *^ *P*<0.05 vs. the Ang II group.

In response to Ang II stimulation, VSMCs acquired strong capacity of proliferation when compared to the non-stimulated cells. Sodium ferulate reduced this increase at three concentrations, and 200 µmol/L sodium ferulate performed the best effect (Figure 1A and Figure S1). Consisted with the results of CCK-8 test, Ang II-treated cells presented approximately two-fold migratory effect to the control. Pre-treatment with sodium ferulate decreased the total number of migratory cells significantly, less than one fifth of the stimulated group (Figure 1B).

### Sodium ferulate attenuates oxidative stress under Ang II stimulation

**Figure 2:**
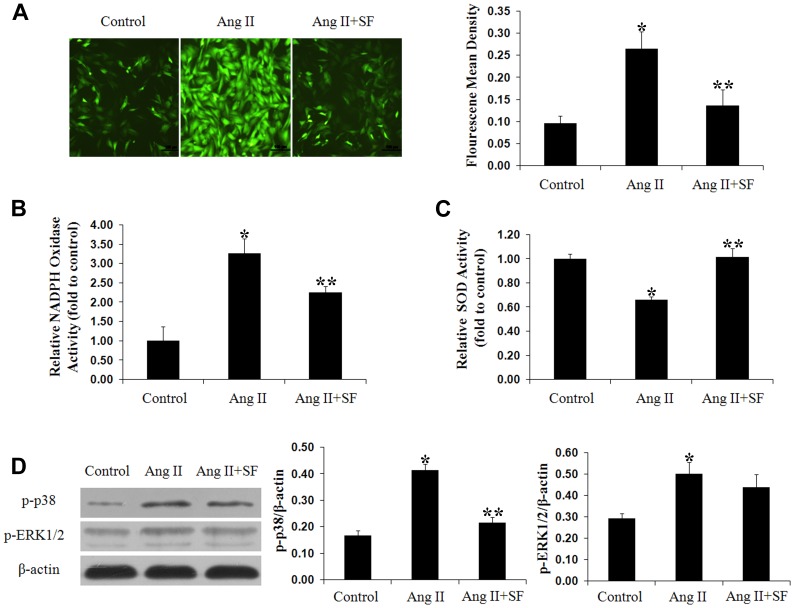
Sodium ferulate attenuates Ang II-induced oxidative stress in VSMCs. After synchronization, VSMCs were stimulated with 1 µmol/L Ang II for 1 hour, with or without sodium ferulate at a dose of 200 µmol/L. (A) Intracellular ROS generation was detected using DCFH-DA probe (× 100 magnifications). NAPDH oxidase (B) and SOD activity (C) were analyzed using ELISA kits. Protein levels of p-p38 and p-ERK1/2 (D) were examined by western blot. N = 3 for each group, ^*^ *P*<0.05 vs. the control group, ^* *^ *P*<0.05 vs. the Ang II group.

Exposed to Ang II for 1 hour, the intracellular ROS level was markedly increased compared to the negative control group. Sodium ferulate inhibited the activation induced by Ang II, and less than half of stimulated cells were identified with ROS expression (Figure 2A). NADPH oxidase, the main source of ROS production [26], was also inhibited by sodium ferulate at a small dose (Figure 2B). Meanwhile, the SOD activity was elevated by sodium ferulate in response to Ang II stimulation (Figure 2C). In present study, p38 MAPK was detected as the potential mechanism explaining for the underlying inhibitory effect of sodium ferulate on oxidative stress. The western blot results demonstrated the Ang II-induced p38 phosphorylation was blocked by sodium ferulate, but no significant difference of ERK1/2 phosphorylation was found (Figure 2D).

### Sodium ferulate upregulates SM α-actin and SM-MHC protein expression under 10% serum stimulation

**Figure 3:**
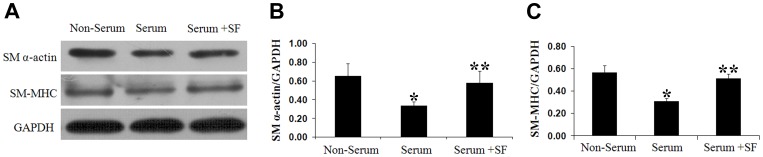
Sodium ferulate upregulates contractile markers expression in response to 10% serum. Cultured VSMCs were pre-treated with serum starvation for 72 hours and then challenged by 10% serum for another 48 hours to induce synthetic VSMCs, with or without 200 µmol/L sodium ferulate. (A) Representative Immunoblots. (B) Relative expression of SM α-actin protein. (C) Relative expression of SM-MHC protein. N = 3 for each group, ^*^ *P*<0.05 vs. the non-serum group, ^* *^ *P*<0.05 vs. the serum group.

Contractile proteins of SM α-actin and SM-MHC in VSMCs were induced during serum starvation, while such feature was alleviated by 10% serum. Pretreatment with Sodium ferulate prevented the decrease caused by serum and reversed both SM α-actin and SM-MHC expression to the levels before serum stimulation (Figure 3).

### Sodium ferulate downregulates Notch and Wnt pathway in response to serum

**Figure 4:**
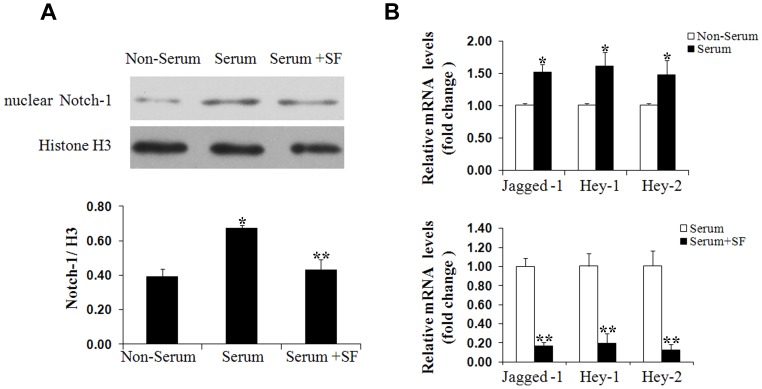
Sodium ferulate inhibits Notch pathway transduction. After serum starvation for 72 hours, VSMCs were exposed to 10% serum for another 48 hours in presence or absence of sodium ferulate at a dose of 200 µmol/L. The nuclear Notch-1 protein (A), the mRNA levels of Jagged-1, Hey-1 and Hey-2 (B) were measured using Western blot and real-time PCR, respectively. N = 3 for each group, ^*^ *P*<0.05 vs. the non-serum group, ^* *^ *P*<0.05 vs. the serum group.

In order to understand the possible mechanism accounting for the protective effects of sodium ferulate on VSMCs phenotype switching, the Notch and Wnt signaling transduction were observed under serum stimulation. As expected, the nuclear Notch-1 protein, Jagged-1, Hey-1, Hey-2 mRNA were increased significantly by 10% serum when compared to the unstimulated group. Sodium ferulate reversed these effects and decreased Notch-1 to 0.64-fold and Jagged-1, Hey-1 and Hey-2 mRNA production by more than 5-fold, 4-fold and 7-fold to the simulated group, respectively (Figure 4).

**Figure 5:**
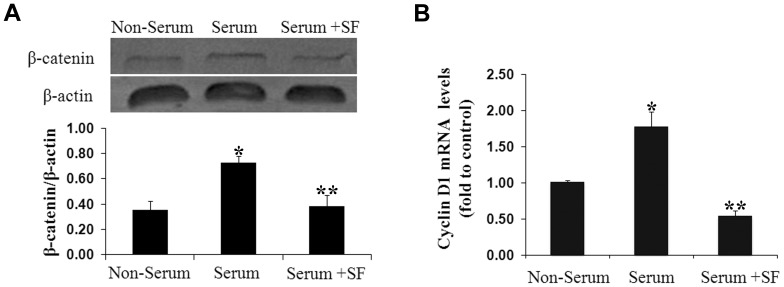
Sodium ferulate blocks activity of Wnt pathway. After serum starvation for 72 hours, VSMCs were exposed to 10% serum for another 48 hours in presence or absence of sodium ferulate at a dose of 200 µmol/L. (A) Total β-catenin protein was detected by Western blot. (B) Cyclin D1 mRNA was measured by real-time PCR. N = 3 for each group, ^*^ *P*<0.05 vs. the non-serum group, ^* *^ *P*<0.05 vs. the serum group.

Similar with the Notch pathway, Wnt pathway components containing β-catenin and Cyclin D1 were also greatly blocked by sodium ferulate under serum induction. In sodium ferulate group, total β-catenin protein and Cyclin D1 mRNA were 0.53-fold and 0.31-fold to the serum group (Figure 5).

### Sodium ferulate inhibits oxidative stress in vivo

8-iso-PGF2α, an indicator of oxidative stress in vivo [27], was evaluated 7 days after artery injury. Compared to the sham group, the serum level of 8-iso-PGF2α was increased significantly after exposure to balloon catheter insertion. In contrast, sodium ferulate decreased 8-iso-PGF2α expression by more than 2-fold compared to the saline group (Table 2).

**Table 2:** Sodium Ferulate reduces serum 8-iso-PGF2α levels.

	Sham group	Saline group	SF group
8-iso-PGF2α (pg/ml)	70.65±53.74	384.69±124.17*	128.16±61.53^**^

Values are expressed as mean ± SD; n = 12 per group.

* *P*<0.05 vs. the sham group, ^* *^ *P*<0.05 vs. the saline group.

### Sodium ferulate enhances contractile markers expression and attenuates collagen generation in injured arteries

**Figure 6:**
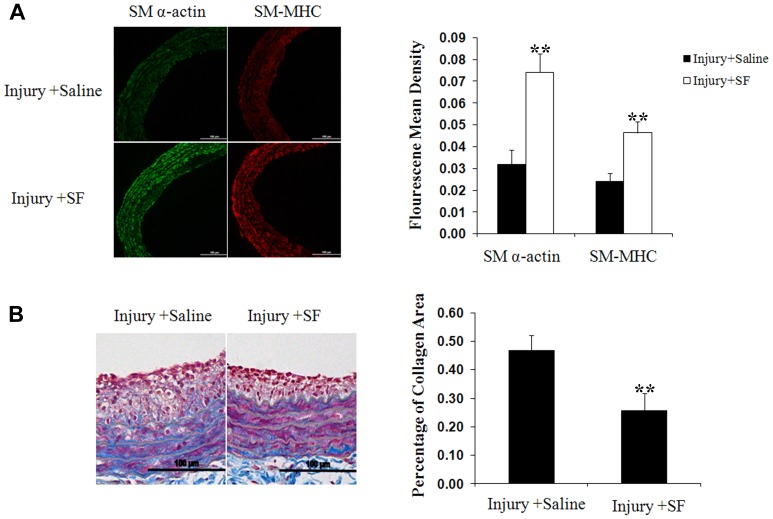
Sodium ferulate increases contractile markers expression and reduces collagen generation in injured arteries. (A) Representative artery sections with immunofluoresence staining for SM α-actin and SM-MHC (n = 12). Scale bar represents 100 µm. (C) Representative Masson trichrome stained vessel sections (n = 12). Collagen displayed as the blue performance and calculated as the percentage of collagen area to intimal and medial area. Scale bar represents 100 µm. *P < 0.05 vs sham group; **P < 0.05 vs saline group.

As indicated above, VSMCs in synthetic state lose contractile ability as well as lowered expression of contractile makers [7]. Sodium ferulate suppressed this decrease, and increased the SM α-actin and SM-MHC expression in arteries. Compared to the saline group, vessels in sodium ferulate group presented much stronger fluorescence throughout the wall (Figure 6A). In addition, the greater synthesis induced by catheter insertion was also blocked by sodium ferulate. The results of Masson staining showed the collagen composition in sodium ferulate group was much less than saline group (Figure 6B).

### Sodium ferulate inhibits neointimal hyperplasia induced by balloon injury

**Figure 7:**
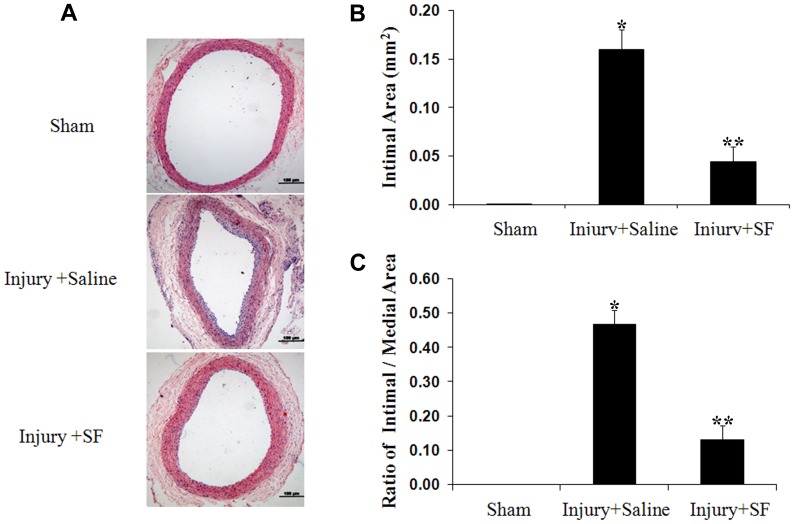
Sodium ferulate suppresses neointimal hyperplasia at 7 days after artery injury. (A) Representative hematoxylin-eosin stained carotid artery sections from each group (n = 12). Scale bar represents 100 µm. Neointimal area (B) and the ratio of intimal area to medial area (C) were measured. *P < 0.05 vs sham group; **P < 0.05 vs saline group.

As results of histomorphological detection shown, a layer of loose neointima close to the internal elastic membrane was generated and the perfusion lumen area was lost markedly in arteries of saline group when compared to the sham group. However, the deterioration on vessels with catheter intervention was alleviated by sodium ferulate. The neointimal formation in sodium ferulate administration group was thinner and intimal cells arranged more orderly than the saline group (Figure 7A). In addition to the smoother intimal surface, the intimal area and ratio of intimal area to medial area were both decreased (Figure 7B, 7C).

## Discussion

In the present study, we demonstrated for the first time that sodium ferulate could inhibit neointimal hyperplasia after artery injury. This conclusion was established based on the observations below. First, sodium ferulate decreased Ang II-induced VSMCs proliferation and migration, which was attributed to significant reduction of excessive ROS generation. Second, sodium ferulate blocked VSMCs phenotypic switching and promoted the synthetic alteration to the contractile phenotype. In addition, the accompanied modulation on molecular mechanism, including inhibition of p38 MAPK, Notch and Wnt signaling were found.

Ang II, a most well-known active peptide of renin-angiotensin system, is also a multiple modulator involved in vasoconstriction, inflammation, thrombosis and vascular remodeling [28]. In response to arterial injury, Ang II is found highly expressed in lesions and plays as an important mediator to trigger oxidative stress and cell growth [29], [30]. In this study, our data confirmed Ang II induced significant increase of ROS in cultured VSMCs, while sodium ferulate inhibited Ang II-induced ROS generation, and attenuated VSMCs proliferation and migration. However, the relationship between sodium ferulate and oxidative stress in VSMCs remains unknown. According to previous studies, imbalance between ROS and anti-oxidants results in oxidative damage [31]. Combination of Ang II to Ang II type 1 receptor stimulates non-phagocytic NADPH oxidase, including NOX1 and NOX4 in VSMCs [32], [33], [34]. The activated NOX accept electrons donated by NADPH and converts molecular oxygen (O_2_) into unstable superoxide state (·O^2−^) and hydrogen peroxide (H_2_O_2_) [35]. These emerging oxidants are regulated by anti-oxidants such as SODs, catalase, heme oxygenase-1, glutathione and other molecules [26]. Sodium ferulate not only decreased NADPH oxidase activity, but also up-regulated total SOD, the important anti-oxidant substance, contributing to sequential suppression of ROS production. In addition, this inhibitory effect was also confirmed in vivo. Rats administrated with sodium ferulate displayed a much lower serum level of 8-iso-PGF2α, which indicated oxidative stress triggered by intimal injury was interrupted by sodium ferulate.

Accumulating evidence suggests ROS participate in a variety of signaling transduction as second messengers [36]. The regulation of ROS on signal pathway is dependent on oxidative modification of target proteins. Generally, these proteins contain redox-reactive cysteine (Cys) residues, which can be oxidized into forms of sulfenic acid (−SOH) or other further oxidative products [37]. Mitogen activated protein kinases (MAPK), as the major targets of ROS, are regulated by Ang II in many cell types [38]. There are two ways for ROS to modify MAPK activity. MAPK phosphatases and protein tyrosine phosphatases share a conserved redox-sensitive cysteine residue, both of which facilitate the turn of MAPK into active state through oxidative modification by ROS [39], [40]. Our data implied p38 and ERK1/2 phosphorylation increased along with aggravation of intracellular ROS under Ang II stimulation, while sodium ferulate blocked p38 activation with no effect on ERK1/2. However, the relationship between sodium ferulate and MAPK pathway lacks of adequate experimental evidence. In rat hippocampus, sodium ferulate suppressed p38 activation, but enhanced ERK1/2 phosphorylation under amyloid-beta stimulation [41]. In human vascular endothelial cells, ferulate acid inhibited JNK rather than p38 and ERK1/2 when exposed to radiation [42]. However, ferulate acid attenuated activation of ERK1/2 and JNK rather than p38 in VSMCs after stimulation with 1 µM Ang II for 15min [43]. Accordingly, the regulation of sodium ferulate on MAPK is dependent on cell type, drug dose, stimulus, stimulation time and other factors.

Previous findings revealed VSMCs could alter from contractile to synthetic state when exposed to various environmental factors, for instance, growth factor, cytokines, hypoxia, injury and mechanical force [44]. The synthetic type cells not only down-regulate contractile protein, including SM α-actin, SM-MHC, calpolin, myocardin and smoothenin, but also acquire strong capacity of synthesis [7]. Collagen, the main component of ECM, is an important product of dedifferentiated VSMCs [45]. In the present study, serum, a known potent stimulator of phenotypic switching on VSMCs [46], induced much lower expression of SM α-actin and SM-MHC. Sodium ferulate blocked this deterioration and up-regulated them as well as in balloon injured arteries. Moreover, the collagen content in neointimal was also decreased by sodium ferulate. Our findings above suggested sodium ferulate attenuated synthetic VSMCs activation induced by vessel injury, which played a pivotal role in inhibition of sequential neointimal hyperplasia. However, the effect of sodium ferulate on vascular relaxation is not clear. Although ferulate acid was considered as an inactive component to induce vascular relaxation in Radix Angelica [47], Chen et al. have demonstrated sodium ferulate is a non-selective relaxant to VSMCs [48]. Unfortunately, we have not made any relevant studies on this issue. Protective relaxation after endothelium deprivation plays a critical role in vascular health, and the relationship between sodium ferulate and vascular relaxation should be investigated in the future study.

The transcriptional pathway involved in regulating VSMCs specific gene contains GATA-6, serum response factor (SRF) and myocardin [49]. Recently, Notch has been found to be an additional pathway critical for regulating VSMCs phenotype [49]. Generally, interaction of Notch ligands (Jagged 1 and 2; Delta-like 1, 3 and 4) and receptors (Notch 1 to 4) induces Notch intracellular domain (NICD) cleavage from membrane and translocation into nucleus, further leading to transcription activation of the effect genes (Hes, Hey) [50]. Sodium ferulate may decrease proteolytical release of NICD through down-regulation of Notch 1 and Jagged 1, which ultimately caused inhibition of Hey-1 and Hey-2 expression. Previous studies suggest Notch signaling participates in regulating VSMCs differentiation and identify SM α-actin and SM-MHC as targets of Notch [51], [52]. Indeed, Jagged 1-Notch interactions can induce SM α-actin expression via NCID/CBF-1 complex binding to specific region of SM α-actin promoter [51]. Over-expression of Hey-1 and Hey-2 turns off this signal by interfering the binding process rather than interruption on NCID/CBF-1 complex [53]. In this way, the decline of Hey-1 and Hey-2 caused by sodium ferulate, relieved inhibition on binding of NCID/CBF-1 complex and marker gene, contributing to increase of SM α-actin and SM-MHC expression.

Wnt pathway is another emerging spot on modulation of VSMCs phenotype alteration. Following with ligands association with receptors, the activation of Wnt signaling facilities target genes expression, which includes Cyclin D1, cMyc and insulin growth factor [54]. Since the decrease of Cyclin D1 dependent on β-catenin/TCF axis, sodium ferulate attenuated cell cycle activation and arrested VSMCs proliferation. In addition, inactive Wnt pathway mediated lower matrix matalloproteinase activity and less ECM composition [55], which contributes to suppression of neointimal generation. Considerable observations have shown that there are several intersections between Notch and Wnt pathway. Here, we focus on three of them, which would involve in sodium ferulate simultaneous inhibition on Notch and Wnt pathway in our study. First, Delta-like-1(Dll-1), a ligand of Notch pathway, is also a downstream gene of Wnt [56]. Sodium ferulate inhibited Wnt signaling activation would down-regulate the Dll-1, which resulted in depression of Notch components. Second, NICD can attach with Dishevelled (Dsh) and thus interrupt Wnt signals as well as Notch pathway itself [57]. Sodium ferulate may promote this combination, so as to enable inactivation of Notch and Wnt transduction. Third, glycogen synthase kinase-3β (GSK-3β) can bind and phosphorylate Notch, inducing NICD degradation [58]. Sodium ferulate could intensify GSK-3β, which caused β-catenin decline and Notch transcriptional arrest. Hence, we deducted that crosstalk between Wnt and Notch pathway would be an important part of complex regulatory networks sodium ferulate acts on VSMCs behavior.

In summary, sodium ferulate could attenuate neointimal hyperplasia after artery insult through blockade of oxidative stress and VSMCs phenotypic switching. The regulatory action involved with complex molecular mechanism, such as MAPK, Notch and Wnt pathway. Based on observations of the beneficial effect showed in this study, sodium ferulate might be a potent therapeutic approach to improve vascular function, and reduce the occurrence of restenosis after PCI.

## Supporting Information

Figure S1 **Effect of sodium ferulate on Ang II- induced VSMCs proliferation.** VSMCs were pre-incubated with sodium ferulate at dose of 200 and 300 µmol/L for 1 hour and stimulated with 1 µmol/L Ang II for 48 hours. Following addition with CCK-8 reagent, OD values were measured at 450 nm (n = 6). ^*^ *P*<0.05 vs. the control group, ^* *^ *P*<0.05 vs. the Ang II group.
(TIF)

## References

[1] Kibos A, Campeanu A, Tintoiu I (2007) Pathophysiology of coronary artery in-stent restenosis. Acute Card Care 9: 111–119. 17573586 10.1080/17482940701263285

[2] Khan W, Farah S, Domb AJ (2012) Drug eluting stents: developments and current status. J Control Release 161: 703–712. 22366546 10.1016/j.jconrel.2012.02.010

[3] Alahmar AE, Grayson AD, Andron M, Egred M, Roberts ED, et al (2009) Reduction in mortality and target-lesion revascularisation at 2 years: a comparison between drug-eluting stents and conventional bare-metal stents in the "real world". Int J Cardiol 132: 398–404. 18439692 10.1016/j.ijcard.2008.03.001

[4] Papafaklis MI, Chatzizisis YS, Naka KK, Giannoglou GD, Michalis LK (2012) Drug-eluting stent restenosis: effect of drug type, release kinetics, hemodynamics and coating strategy. Pharmacol Ther 134: 43–53. 22212618 10.1016/j.pharmthera.2011.12.006

[5] De Labriolle A, Bonello L, Lemesle G, Steinberg DH, Roy P, et al (2009) Clinical presentation and outcome of patients hospitalized for symptomatic in-stent restenosis treated by percutaneous coronary intervention: comparison between drug-eluting stents and bare-metal stents. Arch Cardiovasc Dis 102: 209–217. 19375675 10.1016/j.acvd.2009.01.004

[6] Nikol S, Huehns TY, Hofling B (1996) Molecular biology and post-angioplasty restenosis. Atherosclerosis 123: 17–31. 8782834 10.1016/0021-9150(96)05807-8

[7] Zargham R (2008) Preventing restenosis after angioplasty: a multistage approach. Clin Sci (Lond) 114: 257–264. 18194134 10.1042/CS20070228

[8] Fernandez-Ortiz A, Badimon JJ, Falk E, Fuster V, Meyer B, et al (1994) Characterization of the relative thrombogenicity of atherosclerotic plaque components: implications for consequences of plaque rupture. J Am Coll Cardiol 23: 1562–1569. 8195515 10.1016/0735-1097(94)90657-2

[9] Gibbons GH, Dzau VJ (1994) The emerging concept of vascular remodeling. N Engl J Med 330: 1431–1438. 8159199 10.1056/NEJM199405193302008

[10] Kochiadakis GE, Arfanakis DA, Marketou ME, Skalidis EI, Igoumenidis NE, et al (2010) Oxidative stress changes after stent implantation: a randomized comparative study of sirolimus-eluting and bare metal stents. Int J Cardiol 142: 33–37. 19168247 10.1016/j.ijcard.2008.12.105

[11] Chen Y, Jiang J, Miao H, Chen X, Sun X, et al (2013) Hydrogen-rich saline attenuates vascular smooth muscle cell proliferation and neointimal hyperplasia by inhibiting reactive oxygen species production and inactivating the Ras-ERK1/2-MEK1/2 and Akt pathways. Int J Mol Med 31: 597–606. 23340693 10.3892/ijmm.2013.1256

[12] Zhu L, Hao Y, Guan H, Cui C, Tian S, et al (2013) Effect of sinomenine on vascular smooth muscle cell dedifferentiation and neointima formation after vascular injury in mice. Mol Cell Biochem 373: 53–62. 23065380 10.1007/s11010-012-1474-9

[13] Miyahara T, Runge S, Chatterjee A, Chen M, Mottola G, et al (2013) D-series resolvin attenuates vascular smooth muscle cell activation and neointimal hyperplasia following vascular injury. FASEB J 27: 2220–2232. 23407709 10.1096/fj.12-225615 PMC3659350

[14] Merlet E, Atassi F, Motiani RK, Mougenot N, Jacquet A, et al (2013) miR-424/322 regulates vascular smooth muscle cell phenotype and neointimal formation in the rat. Cardiovasc Res 98: 458–468. 23447642 10.1093/cvr/cvt045 PMC3656613

[15] Chen L, Qi J, Chang YX, Zhu D, Yu B (2009) Identification and determination of the major constituents in Traditional Chinese Medicinal formula Danggui-Shaoyao-San by HPLC-DAD-ESI-MS/MS. J Pharm Biomed Anal 50: 127–137. 19411155 10.1016/j.jpba.2009.03.039

[16] Gao L, He Y, Tang J, Yin J, Huang Z, et al (2013) Genetic Variants of Pregnane X Receptor (PXR) and CYP2B6 Affect the Induction of Bupropion Hydroxylation by Sodium Ferulate. PLoS One 8: e62489. 23840296 10.1371/journal.pone.0062489 PMC3686783

[17] Wang BH, Ou-Yang JP (2005) Pharmacological actions of sodium ferulate in cardiovascular system. Cardiovasc Drug Rev 23: 161–172. 16007232 10.1111/j.1527-3466.2005.tb00163.x

[18] Koh PO (2013) Ferulic acid attenuates the injury-induced decrease of protein phosphatase 2A subunit B in ischemic brain injury. PLoS One 8: e54217. 23349830 10.1371/journal.pone.0054217 PMC3547913

[19] Zhang D, Bi Z, Li Y, Zheng H, Li L, et al (2009) Sodium ferulate modified gene expression profile of oxidized low-density lipoprotein-stimulated human umbilical vein endothelial cells. J Cardiovasc Pharmacol Ther 14: 302–313. 19837969 10.1177/1074248409347986

[20] Choi R, Kim BH, Naowaboot J, Lee MY, Hyun MR, et al (2011) Effects of ferulic acid on diabetic nephropathy in a rat model of type 2 diabetes. Exp Mol Med 43: 676–683. 21975281 10.3858/emm.2011.43.12.078 PMC3256295

[21] Kim SR, Daily JR, Lee YE (2013) Ferulic acid: a novel inhibitor of presynaptic glutamate release. J Med Food 16: 95. 23402635 10.1089/jmf.2013.1602.com

[22] Jin Y, Yan EZ, Fan Y, Guo XL, Zhao YJ, et al (2007) Neuroprotection by sodium ferulate against glutamate-induced apoptosis is mediated by ERK and PI3 kinase pathways. Acta Pharmacol Sin 28: 1881–1890. 18031600 10.1111/j.1745-7254.2007.00634.x

[23] Barone E, Calabrese V, Mancuso C (2009) Ferulic acid and its therapeutic potential as a hormetin for age-related diseases. Biogerontology 10: 97–108. 18651237 10.1007/s10522-008-9160-8

[24] Huang HC, Jan TR, Yeh SF (1992) Inhibitory effect of curcumin, an anti-inflammatory agent, on vascular smooth muscle cell proliferation. Eur J Pharmacol 221: 381–384. 1426014 10.1016/0014-2999(92)90727-l

[25] Sorescu D, Somers MJ, Lassegue B, Grant S, Harrison DG, et al (2001) Electron spin resonance characterization of the NAD(P)H oxidase in vascular smooth muscle cells. Free Radic Biol Med 30: 603–612. 11295358 10.1016/s0891-5849(00)00507-4

[26] Levonen AL, Vahakangas E, Koponen JK, Yla-Herttuala S (2008) Antioxidant gene therapy for cardiovascular disease: current status and future perspectives. Circulation 117: 2142–2150. 18427144 10.1161/CIRCULATIONAHA.107.718585

[27] Basu S (2010) Bioactive eicosanoids: role of prostaglandin F(2alpha) and F(2)-isoprostanes in inflammation and oxidative stress related pathology. Mol Cells 30: 383–391. 21113821 10.1007/s10059-010-0157-1

[28] Dzau VJ (2001) Theodore Cooper Lecture: Tissue angiotensin and pathobiology of vascular disease: a unifying hypothesis. Hypertension 37: 1047–1052. 11304501 10.1161/01.hyp.37.4.1047

[29] Rakugi H, Kim DK, Krieger JE, Wang DS, Dzau VJ, et al (1994) Induction of angiotensin converting enzyme in the neointima after vascular injury. Possible role in restenosis. J Clin Invest 93: 339–346. 8282805 10.1172/JCI116965 PMC293775

[30] Griendling KK, Minieri CA, Ollerenshaw JD, Alexander RW (1994) Angiotensin II stimulates NADH and NADPH oxidase activity in cultured vascular smooth muscle cells. Circ Res 74: 1141–1148. 8187280 10.1161/01.res.74.6.1141

[31] Landmesser U, Harrison DG (2001) Oxidative stress and vascular damage in hypertension. Coron Artery Dis 12: 455–461. 11696684 10.1097/00019501-200109000-00004

[32] Griendling KK, Sorescu D, Ushio-Fukai M (2000) NAD(P)H oxidase: role in cardiovascular biology and disease. Circ Res 86: 494–501. 10720409 10.1161/01.res.86.5.494

[33] Bengtsson SH, Gulluyan LM, Dusting GJ, Drummond GR (2003) Novel isoforms of NADPH oxidase in vascular physiology and pathophysiology. Clin Exp Pharmacol Physiol 30: 849–854. 14678249 10.1046/j.1440-1681.2003.03929.x

[34] Heeneman S, Sluimer JC, Daemen MJ (2007) Angiotensin-converting enzyme and vascular remodeling. Circ Res 101: 441–454. 17761934 10.1161/CIRCRESAHA.107.148338

[35] Babior BM, Lambeth JD, Nauseef W (2002) The neutrophil NADPH oxidase. Arch Biochem Biophys 397: 342–344. 11795892 10.1006/abbi.2001.2642

[36] Forman HJ, Torres M, Fukuto J (2002) Redox signaling. Mol Cell Biochem 234-235: 49–62. 12162460

[37] Ray PD, Huang BW, Tsuji Y (2012) Reactive oxygen species (ROS) homeostasis and redox regulation in cellular signaling. Cell Signal 24: 981–990. 22286106 10.1016/j.cellsig.2012.01.008 PMC3454471

[38] Touyz RM (2004) Reactive oxygen species and angiotensin II signaling in vascular cells — implications in cardiovascular disease. Braz J Med Biol Res 37: 1263–1273. 15273829 10.1590/s0100-879x2004000800018

[39] Thannickal VJ, Fanburg BL (2000) Reactive oxygen species in cell signaling. Am J Physiol Lung Cell Mol Physiol 279: L1005–L1028. 11076791 10.1152/ajplung.2000.279.6.L1005

[40] Lee K, Esselman WJ (2002) Inhibition of PTPs by H(2)O(2) regulates the activation of distinct MAPK pathways. Free Radic Biol Med 33: 1121–1132. 12374624 10.1016/s0891-5849(02)01000-6

[41] Jin Y, Yan EZ, Fan Y, Zong ZH, Qi ZM, et al (2005) Sodium ferulate prevents amyloid-beta-induced neurotoxicity through suppression of p38 MAPK and upregulation of ERK-1/2 and Akt/protein kinase B in rat hippocampus. Acta Pharmacol Sin 26: 943–951. 16038626 10.1111/j.1745-7254.2005.00158.x

[42] Ma ZC, Hong Q, Wang YG, Tan HL, Xiao CR, et al (2010) Ferulic acid attenuates adhesion molecule expression in gamma-radiated human umbilical vascular endothelial cells. Biol Pharm Bull 33: 752–758. 20460750 10.1248/bpb.33.752

[43] Hou YZ, Yang J, Zhao GR, Yuan YJ (2004) Ferulic acid inhibits vascular smooth muscle cell proliferation induced by angiotensin II. Eur J Pharmacol 499: 85–90. 15363954 10.1016/j.ejphar.2004.07.107

[44] Davis-Dusenbery BN, Wu C, Hata A (2011) Micromanaging vascular smooth muscle cell differentiation and phenotypic modulation. Arterioscler Thromb Vasc Biol 31: 2370–2377. 22011749 10.1161/ATVBAHA.111.226670 PMC4429757

[45] Frid MG, Dempsey EC, Durmowicz AG, Stenmark KR (1997) Smooth muscle cell heterogeneity in pulmonary and systemic vessels. Importance in vascular disease. Arterioscler Thromb Vasc Biol 17: 1203–1209. 9261247 10.1161/01.atv.17.7.1203

[46] Shimoyama T, Hiraoka S, Takemoto M, Koshizaka M, Tokuyama H, et al (2010) CCN3 inhibits neointimal hyperplasia through modulation of smooth muscle cell growth and migration. Arterioscler Thromb Vasc Biol 30: 675–682. 20139355 10.1161/ATVBAHA.110.203356

[47] Rhyu MR, Kim JH, Kim EY (2005) Radix angelica elicits both nitric oxide-dependent and calcium influx-mediated relaxation in rat aorta. J Cardiovasc Pharmacol 46: 99–104. 15965361 10.1097/01.fjc.0000164092.88821.49

[48] Chen GP, Ye Y, Li L, Yang Y, Qian AB, et al (2009) Endothelium-independent vasorelaxant effect of sodium ferulate on rat thoracic aorta. Life Sci 84: 81–88. 19038273 10.1016/j.lfs.2008.11.003

[49] Morrow D, Guha S, Sweeney C, Birney Y, Walshe T, et al (2008) Notch and vascular smooth muscle cell phenotype. Circ Res 103: 1370–1382. 19059839 10.1161/CIRCRESAHA.108.187534

[50] Artavanis-Tsakonas S, Rand MD, Lake RJ (1999) Notch signaling: cell fate control and signal integration in development. Science 284: 770–776. 10221902 10.1126/science.284.5415.770

[51] Noseda M, Fu Y, Niessen K, Wong F, Chang L, et al (2006) Smooth Muscle alpha-actin is a direct target of Notch/CSL. Circ Res 98: 1468–1470. 16741155 10.1161/01.RES.0000229683.81357.26

[52] Doi H, Iso T, Sato H, Yamazaki M, Matsui H, et al (2006) Jagged1-selective notch signaling induces smooth muscle differentiation via a RBP-Jkappa-dependent pathway. J Biol Chem 281: 28555–28564. 16867989 10.1074/jbc.M602749200

[53] Tang Y, Urs S, Liaw L (2008) Hairy-related transcription factors inhibit Notch-induced smooth muscle alpha-actin expression by interfering with Notch intracellular domain/CBF-1 complex interaction with the CBF-1-binding site. Circ Res 102: 661–668. 18239137 10.1161/CIRCRESAHA.107.165134 PMC2662732

[54] Lyon C, Mill C, Tsaousi A, Williams H, George S (2011) Regulation of VSMC behavior by the cadherin-catenin complex. Front Biosci (Landmark Ed) 16: 644–657. 21196194 10.2741/3711

[55] Mill C, George SJ (2012) Wnt signalling in smooth muscle cells and its role in cardiovascular disorders. Cardiovasc Res 95: 233–240. 22492675 10.1093/cvr/cvs141

[56] Galceran J, Sustmann C, Hsu SC, Folberth S, Grosschedl R (2004) LEF1-mediated regulation of Delta-like1 links Wnt and Notch signaling in somitogenesis. Genes Dev 18: 2718–2723. 15545629 10.1101/gad.1249504 PMC528889

[57] Ramain P, Khechumian K, Seugnet L, Arbogast N, Ackermann C, et al (2001) Novel Notch alleles reveal a Deltex-dependent pathway repressing neural fate. Curr Biol 11: 1729–1738. 11719214 10.1016/s0960-9822(01)00562-0

[58] Espinosa L, Ingles-Esteve J, Aguilera C, Bigas A (2003) Phosphorylation by glycogen synthase kinase-3 beta down-regulates Notch activity, a link for Notch and Wnt pathways. J Biol Chem 278: 32227–32235. 12794074 10.1074/jbc.M304001200

