# Supplementary figures and images for: Sodium Ferulate Inhibits Neointimal Hyperplasia in Rat Balloon Injury Model
Source: PLoS One. 2014 Jan 29;9(1):e87561. doi: 10.1371/journal.pone.0087561 (PMC3906191; doi:10.1371/journal.pone.0087561)

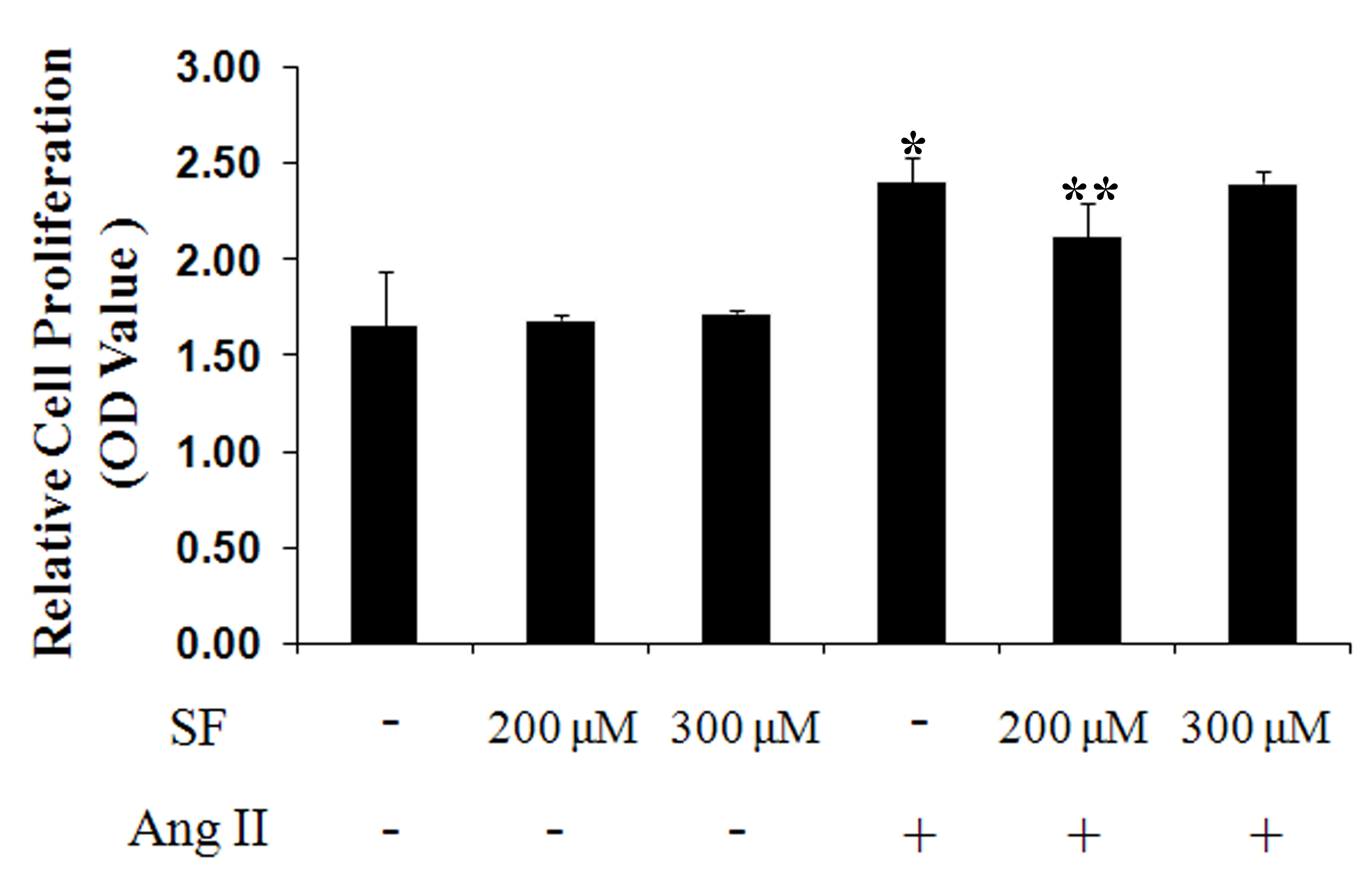

Supplement: Figure S1 — Effect of sodium ferulate on Ang II- induced VSMCs proliferation. VSMCs were pre-incubated with sodium ferulate at dose of 200 and 300 µmol/L for 1 hour and stimulated with 1 µmol/L Ang II for 48 hours. Following addition with CCK-8 reagent, OD values were measured at 450 nm (n = 6). * P<0.05 vs. the control group, * * P<0.05 vs. the Ang II group. (TIF) [file pone.0087561.s001.tif]
